# Which patients miss appointments with general practice and the reasons why: a systematic review

**DOI:** 10.3399/BJGP.2020.1017

**Published:** 2021-05-05

**Authors:** Joanne Parsons, Carol Bryce, Helen Atherton

**Affiliations:** Warwick Medical School, University of Warwick, Coventry, UK.; Warwick Medical School, University of Warwick, Coventry, UK.; Warwick Medical School, University of Warwick, Coventry, UK.

**Keywords:** did not attend, general practice, missed appointments, primary care

## Abstract

**Background:**

Missed GP appointments have considerable time and cost implications for healthcare services.

**Aim:**

This systematic review aims to explore the rate of missed primary care appointments, what the reported reasons are for appointments being missed, and which patients are more likely to miss appointments.

**Design and setting:**

This study reports the findings of a systematic review. The included studies report the rate or reasons of missed appointments in a primary care setting.

**Method:**

Databases were searched using a pre-defined search strategy. Eligible studies were selected for inclusion based on detailed inclusion criteria through title, abstract, and full text screening. Quality was assessed on all included studies, and findings were synthesised to answer the research questions.

**Results:**

A total of 26 studies met the inclusion criteria for the review. Of these, 19 reported a rate of missed appointments. The mean rate of missed appointments was 15.2%, with a median of 12.9%. Twelve studies reported a reason that appointments were missed, with work or family/childcare commitments, forgetting the appointment, and transportation difficulties most commonly reported. In all, 20 studies reported characteristics of people likely to miss appointments. Patients who were likely to miss appointments were those from minority ethnicity, low sociodemographic status, and younger patients (*<*21 years).

**Conclusion:**

Findings from this review have potential implications for targeted interventions to address missed appointments in primary care. This is the first step for clinicians to be able to target interventions to reduce the rate of missed appointments.

## INTRODUCTION

Missed GP appointments have substantial time and cost implications for the NHS. Recent estimates suggest more than 15 million appointments are missed annually in England.^1^ Approximately 7.2 million of these are missed appointments with GPs, costing NHS England £216 million per year.^1^ The high volume of missed appointments exacerbates the increasing demand on GPs and primary care by taking up and not using valuable appointment slots at a time when patients are presenting with more complex and comorbid conditions.^2^ Understanding why patients miss appointments, and how to best manage this, is therefore an important research concern.

Though patients may feel that missed appointments are frustrating for GPs and that receptionists find them annoying, GPs’ views are less negative, and they often consider them as time to catch up.^3^ It is clear, however, that missed appointments can lead to unresolved medical problems, leaving patients vulnerable and presenting later, or living with untreated or worsening health.^4^ Previous research shows that the patients most likely to miss GP appointments are those with multimorbidity,^5^ those living in high deprivation areas,^6^^,^^7^ those with mental health problems^6^ or young adults.^7^ Research looking at why appointments are missed found the most common reasons to be: forgetting appointments, difficulty cancelling appointments, inconvenient appointment time, being too ill to attend, or no longer needing appointments due to resolved health issues.^8^^,^^9^

A systematic review of missed GP appointments was published in 2003;^9^ since then, much has changed in the way that general practice appointments have been delivered and planned, in particular during the COVID-19 pandemic, making a review timely. Interventions and policies to reduce missed appointments have been introduced,^10^ with changes including online triage and booking,^11^ telephone consultations,^12^ and SMS appointment reminders.^13^^,^^14^

This review aims to provide valuable insight to those commissioning and delivering GP services, by examining which patients miss booked appointments in general practice and to examine why this happens.

## METHOD

### Inclusion and exclusion criteria

PRISMA guidelines^15^ were followed for this study. The inclusion criteria for the review were: any study design; studies that examined missed booked routine appointments with staff at general practices (or the equivalent in non-UK studies); studies that included statistical information about rates of missed booked appointments, the reasons appointments are missed, or both, published in English from 2003 onwards.

### Search strategy

MEDLINE, Embase, Web of Science, Scopus, Cochrane Library database, PsycINFO and Cochrane Central Register of Controlled Trials (CENTRAL) were searched. Reference lists of all included studies were also searched. Searches included all terms relevant to the intervention being examined. (see Supplementary Box S1 for an example of the full search strategy used). Searches included records from May 2003, when the previous review of this topic was published, and were run in September 2019. No language restrictions were placed on the searches.

**Table:** How this fits in

Missed GP appointments have considerable time and cost implications for healthcare services. This review reveals how many booked primary care appointments are missed, the reasons given for this, and what characteristics are commonly associated with missed appointments. This has implications for general practices and clinicians aiming to reduce rates of missed appointments, and for implementing strategies for this.

### Screening and selection of studies

After duplicates were removed, titles and abstracts of remaining results were independently screened by two authors against the inclusion criteria. Discrepancies were resolved by a third author. Full texts were retrieved for all studies meeting inclusion criteria at title and abstract stage, and were then subjected to full text screening. Studies that met the inclusion criteria at full text stage were included in the review. Discrepancies with full text inclusion were resolved by a third author.

### Extraction

Data were extracted using a specifically designed form by two authors independently, and any discrepancies were discussed and resolved by a third author, if necessary. Setting, participants, the rate of missed appointments, reasons given for missing appointments, and characteristics of participants missing appointments were extracted from each study.

### Quality assessment

The mixed methods appraisal tool (MMAT) version 2018^16^ was used to assess quality of included studies. An overall quality rating was determined, for contextual information only, based on the number of positive or negative scores each study was rated. This tool is appropriate for use where studies use a range of methodologies, as is the case for this review. Each study was assessed using five assessment points, and then an overall rating system was applied to each study.^17^ Studies were given an overall rating of high quality if four or five criteria were met, moderate quality if three criteria were met, and low quality if two or less criteria were met.^17^

### Data analysis

Rates of missed appointments (percentage of appointments missed, mean and median) were extracted where reported and, when other data on the number of missed appointments were included, the authors calculated a rate. Included studies which reported reasons for missed appointments or characteristics of patients missing appointments were analysed thematically by two authors classifying results, from which themes were derived. Using a narrative synthesis, the authors looked at themes across the data. Narrative synthesis enables studies with different designs to be analysed in a systematic way considering the similarities and differences between the studies.^18^

## RESULTS

A total of 4906 results were screened, resulting in the inclusion of 26 studies in the review.^3^^,^^5^^,^^8^^,^^10^^,^^19^^–^^40^ Screening process and numbers and reasons for exclusions can be found in the PRISMA flowchart in Supplementary Figure S1.^15^

The main characteristics of included studies can be found in Table 1 and Supplementary Table S1. Of the 26 included studies, 19^19^^,^^20^^,^^23^^–^^25^^,^^27^^–^^40^ reported a rate of missed appointments, and two^5^^,^^10^ reported a rate of number of patients that missed appointments. In all, 12 studies^3^^,^^8^^,^^19^^,^^21^^–^^29^ reported reasons that patients miss appointments. Of these, three^3^^,^^21^^,^^26^ reported health care professionals’ opinions on why patients miss appointments, and the remaining nine^8^^,^^19^^,^^22^^–^^25^^,^^27^^–^^29^ presented patient reported reasons for missing appointments. A total of 20 studies^5^^,^^8^^,^^10^^,^^19^^,^^21^^,^^23^^–^^36^^,^^40^ described characteristics of patients that missed appointments.

**Table 1.** Characteristics of included studies

**Characteristics**	**Number of included studies, *n***
**Country**	
US	14 ^10^^,^^22^^–^^23^^,^^25^^,^^27^^,^^29^^–^^32^^,^^34^^–^^37^^,^^40^
UK	6 ^3^^,^^5^^,^^8^^,^^21^^,^^33^^,^^39^
Australia	2^26^^,^^28^
Canada	2^19^^,^^38^
Malaysia	2^20^^,^^24^

**Date study undertaken**	
2004–2009	11 ^3^^,^^8^^,^^10^^,^^20^^–^^22^^,^^24^^,^^27^^,^^30^^,^^34^^,^^37^
2010–2014	8 ^5^^,^^19^^,^^23^^,^^31^^–^^33^^,^^35^^–^^36^
2015–2019	7 ^25^^–^^26^^,^^28^^–^^29^^,^^38^^–^^40^

**Study design**	
Cross-sectional	15 ^5^^,^^10^^,^^19^^,^^24^^–^^25^^,^^27^^–^^31^^,^^33^^,^^34^^,^^36^^,^^38^^–^^39^
Non-randomised controlled trial	5 ^8^^,^^23^^,^^32^^,^^37^^,^^40^
Qualitative	4 ^3^^,^^21^^–^^22^^,^^26^
Randomised controlled trial	2^20^^,^^35^

**Population**	
All registered patients	17 ^5^^,^^19^^,^^20^^,^^22^^,^^24^^,^^27^^–^^33^^,^^35^^,^^37^^–^^40^
Adults (>18 years old)	4 ^8^^,^^10^^,^^25^^,^^34^
Healthcare staff	3 ^3^^,^^21^^,^^26^
Children/adolescents (<21 years old)	2^23^^,^^36^

**Healthcare system**	
Universal healthcare (UK)	6 ^3^^,^^5^^,^^8^^,^^21^^,^^33^^,^^39^
Healthcare which is a mix of government funded and private insurance (majority government funded, Canada, Australia, and Malaysia)	6 ^19^^,^^20^^,^^24^^,^^26^^,^^28^^,^^38^
Private health insurance and public health coverage (majority private insurance, US)	14 ^10^^,^^22^^–^^23^^,^^25^^,^^27^^,^^29^^–^^32^^,^^34^^–^^37^^,^^40^

### Quality assessment

Overall, 25^3^^,^^5^^,^^8^^,^^10^^,^^20^^–^^40^ of the included studies were rated as high quality, while one was rated as moderate quality overall.^19^ All studies stated a clear research question and appropriate study design. The most frequent unmet criterion was: ‘Are confounders accounted for in the design and analysis?’ (a confounder being a variable that influences both the dependent variable and independent variable, causing a spurious association) in quantitative non-randomised studies, with four out of five^8^^,^^23^^,^^32^^,^^40^ failing to satisfy this. Quality assessment ratings for each included study can be found in Supplementary Table S2.

### Rate of missed appointments

In the 19 studies^19^^,^^20^^,^^23^^–^^25^^,^^27^^–^^40^ that reported a rate of missed appointments, the overall rate was between 3.3% and 48.1%, with a mean of 15.2% and a median of 12.9%. Rates of missed appointments were grouped by country of study. The rate of missed appointments was similar between countries (Table 2). One study from Malaysia^20^ had a particularly high rate of missed appointments, at 48.1%. This study looked at a clinic which operated a walk-in system for standard care and booked appointments for follow-up care. The missed appointment rate was for the booked follow-up appointments.

**Table 2.** Rate of missed appointments by country of study

**Country**	**Number of studies reporting rate**	**Rate of missed appointments**
US	12 ^23^^,^^25^^,^^27^^–^^32^^,^^34^^–^^37^^,^^40^	4.4–29.8% (mean 14.5%)
UK	2 ^33^^,^^39^	5.2% and 12.1%
Australia	1^28^	7.6%
Canada	2^19^^,^^38^	24.6% and 3.3%
Malaysia	2^20^^,^^24^	48.1% and 16.7%

Rates of missed appointments were collated by participant group within the study. Participants were categorised by the various studies as adults (*≥*18 years), children/adolescents (*<*21 years) or all patients (all registered patients of a practice). Rates of missed appointments were similar for each group of participants, but studies that reported a rate of missed appointments among patients who were children/adolescents^23^^,^^36^ were slightly higher than rates for adults or all-patients (Table 3).^19^^–^^20^^,^^24^^–^^25^^,^^27^^–^^35^^,^^37^^–^^40^

**Table 3.** Rate of missed appointments by age group of participants

**Age group**	**Number of studies reporting rate**	**Rate of missed appointments**	**Mean rate, if applicable**
Adults	2^25^^,^^34^	16.3–24.6%	19.1%
Children/adolescents	2^23^^,^^36^	20.4% and 29.8%	
All-patients	15 ^19^^–^^20^^,^^24^^,^^27^^–^^33^^,^^35^^,^^37^^–^^40^	4.4–48.1%	13.3%

### Rate of patients missing appointments

Two studies^5^^,^^10^ reported rates of patients missing appointments. Both considered the relationship between patient health and missed appointments. The first of these reported that 73.0% of patients missed one or more appointment during the study period, with the highest rates found in patients with a psychological health diagnosis.^10^ The second reported that 46.7% of patients missed one or more appointment, indicating missed appointments were a significant marker for subsequent all-cause mortality, particularly in those with a long-term mental health condition.^5^

### Reasons for missed appointments

Overall, 12 studies^3^^,^^8^^,^^19^^,^^21^^–^^29^ discussed reasons that appointments were missed, which were categorised into two themes: patient-centred reasons and clinic-specific reasons, each with subthemes identified (Table 4).

#### Patient-centred reasons for missing appointments

This included work or family/childcare commitments, patients forgetting appointments, difficulty with transport to and from the appointment, feeling too ill to attend, barriers relating to weather, or feeling better by the time of the appointment (Table 4).

**Table 4.** Frequency of reasons for missed appointments

**Themes**	**Frequency (how many studies reported the reason), *n***
**Patient-centred reasons**	
Work or family/childcare issues	7 ^3^^,^^8^^,^^19^^,^^21^^–^^24^
Forgot appointment	5 ^8^^,^^19^^,^^21^^,^^23^^,^^25^
Transportation issues	5^8^^,^^19^^,^^21^^–^^23^
Too unwell	3 ^8^^,^^21^^,^^24^
Weather	3 ^8^^,^^19^^,^^21^
Felt better	2^8^^,^^19^
Other issues: couldn’t be bothered, was in hospital, not aware of date, death in family (each mentioned once)	1^8^

**Clinic-specific reasons**	
Doctor–patient relationship issues (including not with preferred GP and doctor reasons)	5^3^^,^^8^^,^^21^^,^^24^^,^^26^	
Issues with booking system	3 ^3^^,^^21^^–^^22^
Miscommunication	3 ^8^^,^^24^^–^^25^
Monday appointment	3 ^27^^–^^29^
Not receiving a reminder	1 ^19^

#### Clinic specific reasons for missing appointments

These were reasons related to practical aspects of the clinic or process and included doctor–patient relationships, appointments not being with a patient’s preferred GP, issues with the practice’s booking system, miscommunication from the practice about appointments (for example, the wrong date or time being put on appointment cards), day of the week (with Mondays being most mentioned), or not receiving appointment reminders (Table 4).

### Healthcare professional views on missed appointments

Three studies^21^^,^^26^^,^^30^ reported healthcare professionals’ opinions on missed appointments, generally mirroring patient reported reasons. These included patients lacking in health knowledge, difficulty in cancelling appointments, issues around the relationship between the patient and GP, and competing priorities from work and family/childcare commitments. Healthcare professionals also reported that patients missed appointments because they felt better or could not be bothered to attend.

### Characteristics of patients missing appointments

Of the studies included in this review, 205,8,10,19,21,23–36,40 reported characteristics of patients missing appointments. These were categorised into two main themes, each with further subthemes (Table 5).

**Table 5.** Characteristics of patients missing appointments

**Characteristic**	**Frequency (how many studies the characteristics were reported), *n***
**Health-related factors**	
Presence of mental health diagnosis	5 ^5^^,^^8^^,^^10^^,^^21^^,^^31^
Presence of at least one physical health condition	4 ^5^^,^^10^^,^^24^^,^^32^

**Demographic-related factors**	
*Socioeconomic status*	
Minority/non-white patients	8 ^23^^–^^25^^,^^27^^–^^28^^,^^31^^–^^32^^,^^34^
Low sociodemographic status/deprived areas	5 ^21^^,^^24^^,^^26^^,^^33^^,^^36^
Poor education	1^26^
*Insurance status*	
Medicaid or self-pay	4 ^10^^,^^25^^,^^27^^,^^30^
Publicly funded insurance	2^23^^–^^24^
*Age*	
Younger patients (<21 years)	8 ^8^^,^^10^^,^^21^^,^^25^^,^^27^^–^^28^^,^^30^^–^^31^^,^^34^
Older patients (>75 years)	2^10^^,^^33^
Patients aged 20–39 years	1^31^
*Sex*	
Female	3 ^28^^,^^31^^,^^33^
Male	2^8^^,^^24^
*Patient status*	
Patients who have previously missed appointments	3 ^8^^,^^29^^,^^35^
New patients	1^30^
*Type of appointment*	
Appointment booked further away	3^14^^,^^19^^,^^32^
Scheduled Well-child appointment	2^23^^,^^36^
Live further away from appointment location	1^36^

#### Health-related factors

Patients with a mental health diagnosis, or with multiple or serious physical health conditions were more likely to miss appointments (Table 5).

#### Demographic-related factors

Patients were more likely to miss booked appointments if they were of lower socioeconomic status or living in a deprived area, or from a non-white or minority ethnicity group, reflecting areas of unmet need among already disadvantaged groups. Patients in receipt of Medicaid (state-funded health-coverage for eligible groups in the US),^41^ or who are paying for their insurance themselves, or who were receiving publicly funded insurance were more likely to miss appointments. Both younger patients (*<*21 years) and older patients (*>*75 years) were frequently reported as more likely to miss appointments. Some studies found females were more likely to miss appointments, while others found that males were more likely to miss appointments.Patients that have previously missed an appointment were reported as being more likely to miss future appointments. Characteristics of appointments that were more frequently missed included scheduled Well-Child appointments, those that were booked further away from the time of booking, and those that were booked at a practice located further away from where the patient lives (Table 5).

## DISCUSSION

### Summary

This review examined the rate of missed general practice appointments, reasons for missing appointments, and which patients were more likely to miss appointments. The 19 studies that reported a rate of missed appointments showed that between 3.3% and 48.1% of appointments were missed, with a mean of 15.2%. The most frequently reported reasons for missed appointments included work or family/childcare commitments, forgetting, and transport or weather difficulties. Patients that were most likely to miss an appointment included those that were younger (*<*21 years), who had missed appointments previously, from low socioeconomic backgrounds, and those with a mental health or physical diagnosis. All but one of the studies received an overall rating of high quality. The authors’ findings echo those of George and Rubin;^9^ though much has changed in the primary care landscape, it does not appear to be reflected in a reduction in rates of missed appointments between 2003 and this review.

### Strengths and limitations

This review’s strength lies in its breadth, as it covers multiple countries and healthcare systems, and all study designs, providing a wide overview of the literature. However, comparing different healthcare systems can be problematic, as rates and reasons for missed appointments are likely to be affected by the differing health care systems in different countries, and differing payment structures and insurance systems.

The different study designs make it difficult to directly compare studies’ results. Furthermore, different appointment systems across studies make direct comparison difficult. In intervention studies, baseline missed appointment rates were used, but interventions may have been implemented where there was a known issue with the missed appointment rate, and so may not be typical. Exploring reasons given for missing appointments by grouping patient types has the potential to miss detail that relates to patient demographics, and the authors’ findings show that patient demographics have an impact on whether they are more likely to miss appointments.

### Comparison with existing literature

Findings from this review are in line with an earlier systematic review that reported similar rates of missed appointments in the US (5–55%) and the UK (2.9–11.7%).^9^ Reasons for missed appointments in the current review were in line with previous literature, including patients forgetting appointments, being too ill to attend,^8^^,^^9^ feeling better, work and family/childcare commitments, and weather and transport problems.^8^

In line with the current review, previous research suggests that younger patients (*<*21 years), those that have previously missed appointments, being of low socioeconomic status, having psychological problems, and being funded by the state or self-paying (Medicaid or self-pay) were more likely to miss appointments.^8^^,^^9^

### Implications for research and practice

This review has highlighted specific clinical issues that could be addressed to reduce missed appointments, including reviewing booking systems and the availability of appointments with preferred clinicians.

Tailoring appointment scheduling to patient behaviours is a potential approach to reducing non-attendance. For example, because patients who miss appointments are more likely to do so on a Monday, they could be encouraged to schedule non-urgent appointments on other days of the week.

The most cited patient-centred reasons for missing appointments relate to patients’ schedules, for example, having to take time off work or finding childcare. Practices may wish to review their access systems, or use the increase in remote consulting to offer a range of options if that better suits their practice population. Simply forgetting the appointment was also highly cited as a reason for missing an appointment and implementing reminder systems such as SMS reminders, which have been shown to work,^42^^,^^43^ would help with this.

Understanding characteristics of individuals most likely to miss appointments is useful in designing and planning appointment systems in general practice. This review highlights particular groups who are more likely to miss appointments, including those with a mental health diagnosis, those with multiple health conditions, those in ethnic minority groups, and those attending practices in areas of high deprivation. Deprivation has been shown to intersect with multimorbidity, mental health conditions, age, and ethnicity,^44^^,^^45^ meaning that some practices will be addressing several factors at once when tackling missed appointments. Any intervention will need to address multiple patient characteristics, which is then likely to have the most impact on non-attendance.

Future research should examine whether consultation type impacts the rate of missed appointments, reflecting the rapid adoption of remote consulting as a response to COVID-19. This should include the impact on different patient groups, particularly those that are both underserved and not attending. It may be that the move to remote consulting impacts on this group differently. Tailored interventions for specific population subgroups with high rates of missed appointments could then be targeted for improvement. In the shorter term, as the pandemic progresses, practices can audit their missed appointment levels and compare these to pre-pandemic levels to look for differences as a result of a change in access systems.

Future research needs to consider differences in missed appointments between initial consultations and follow-up appointments, and the potential impact of relational continuity on attendance.

This review is of research conducted pre-COVID-19 and it would be useful after the pandemic to consider changes made to the booking of appointments during the pandemic, and whether this has had any impact on missed appointments as compared to before and during the pandemic.

Many studies did not clearly report on practice appointment booking systems; therefore, this needs to be the focus of future research to understand its impact on missed appointment rates. Changes in appointment delivery, accelerated by COVID-19, should be examined to understand their impact on missed appointments. To do this, it needs to be explored with patients why they miss appointments, and established what would work to encourage them to cancel or attend appointments. Qualitative interviews with patients who both miss and do not miss appointments would be beneficial in achieving this aim.
